# Supplementary material for: Predicting the HER2 status in oesophageal cancer from tissue microarrays using convolutional neural networks
Source: Br J Cancer. 2023 Jan 30;128(7):1369–76. doi: 10.1038/s41416-023-02143-y (PMC10050393; doi:10.1038/s41416-023-02143-y)

**Supplemental Material**

**Predicting the HER2 status in esophageal cancer from tissue microarrays using convolutional neural networks**

Juan I. Pisula, Rabi R. Datta, Leandra Börner Valdez, Jan-Robert Avemarg, Jin-On Jung, Patrick Plum, Heike Löser, Philipp Lohneis, Monique Meuschke, Daniel Pinto dos Santos, Florian Gebauer, Alexander Quaas, Christiane J. Bruns, Axel Walch, Kai Lawonn, Felix C. Popp and Katarzyna Bozek

**Supplemental Table 1**. Staining patterns used by pathologists to assess the IHC score of HER2 stainings in biopsies. This analysis method was used because TMAs resemble biopsies more than whole slides.

| **IHC score** | **Pattern of IHC staining for HER2** |  | **HER2 status** |
| --- | --- | --- | --- |
| **0** | No reactivity or membranous reactivity in any (or <5) tumor cell(s) |  | ***negative*** |
| **1** | Tumor cell cluster with a very weak membranous reactivity (at least 5 tumor cells) |  | ***negative*** |
| **2** | Tumor cell cluster with a weak to moderate complete, basolateral or lateral only membranous reactivity (at least 5 tumor cells) |  | ***equivocal* (ISH assessment required)** |
| **3** | Tumor cell cluster with a strong complete, basolateral or lateral only membranous reactivity (at least 5 tumor cells) |  | ***positive*** |

**Supplemental Figure 1**. IHC score distribution of our in-house datasets (with score 2 separated by positive and negative HER2 status).

**
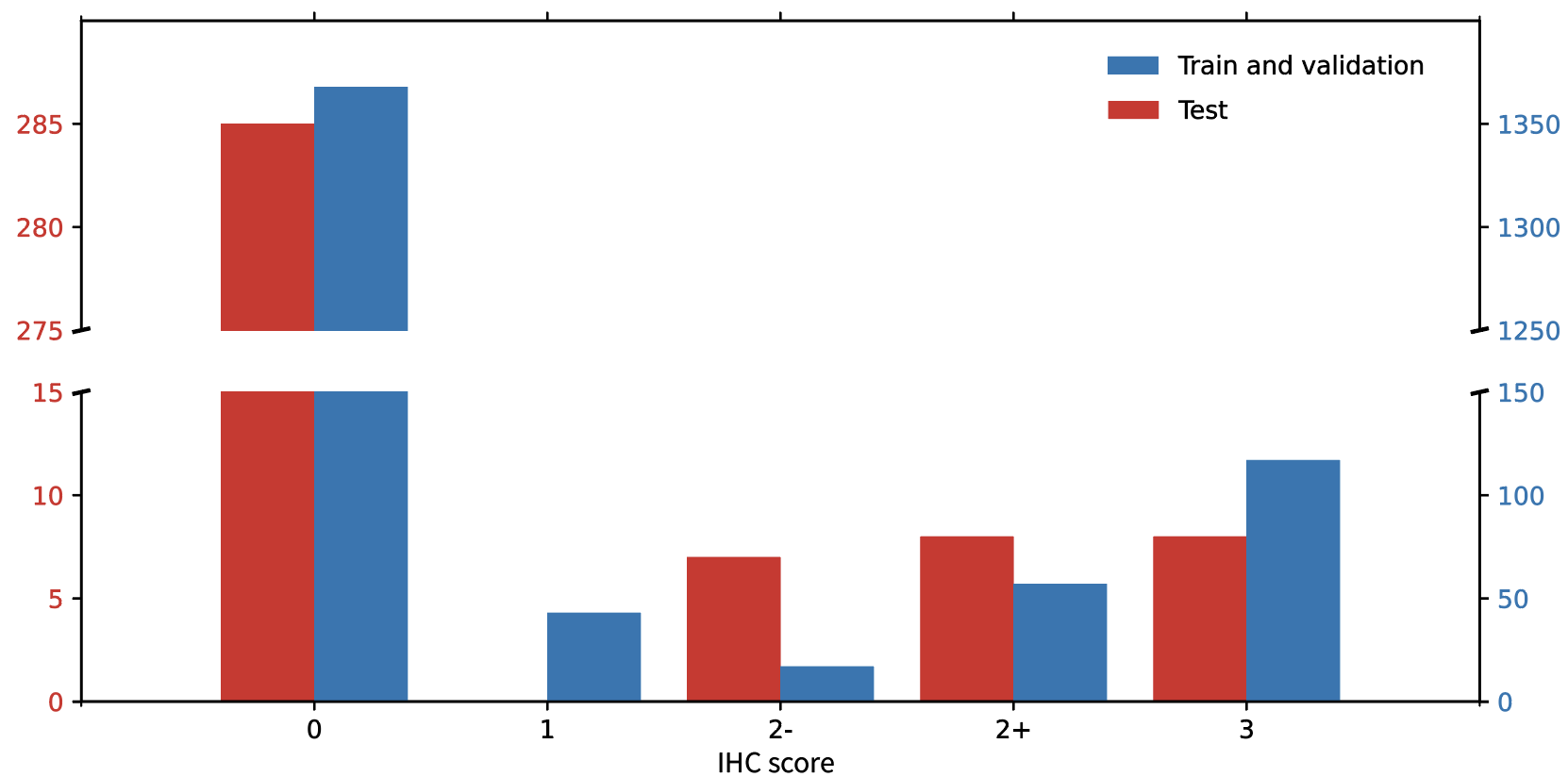
**

**Supplemental Figure 2**. High attention regions indicated in Figure 3. The region of the image is enlarged, the highest attention area is marked with a contour. The area contains elongated tumor cells with only faint staining.


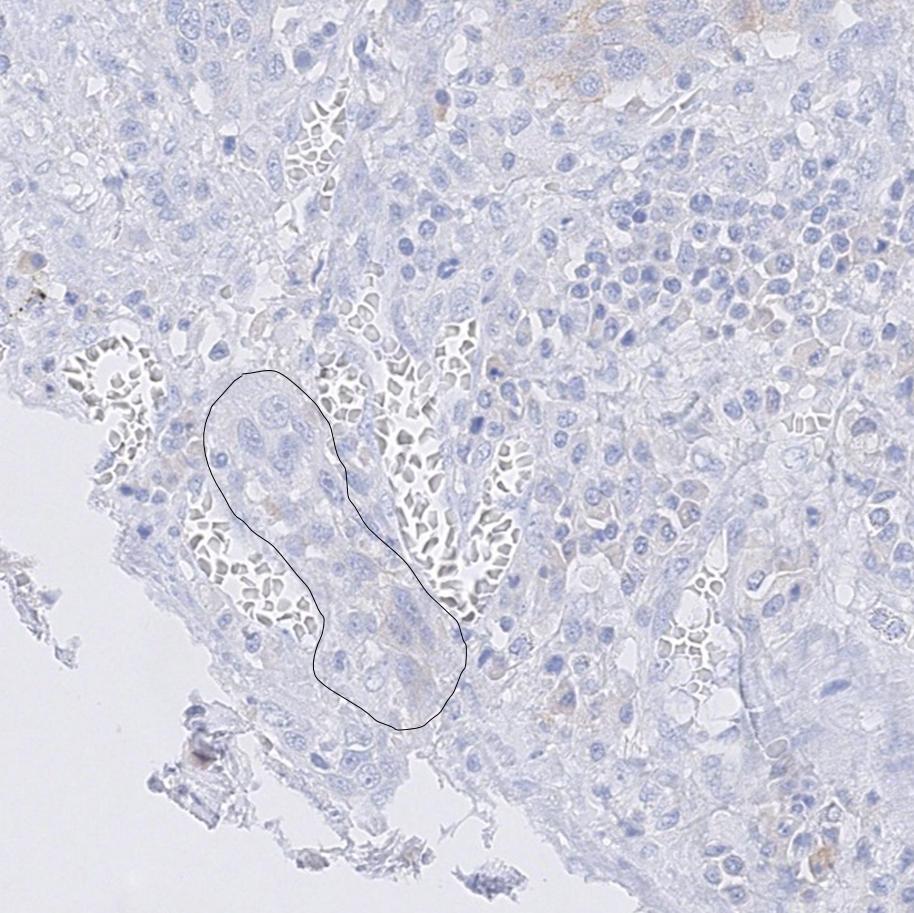

Supplement: Supplementary file 1 — Supplemental material [file 41416_2023_2143_MOESM1_ESM.docx]
